# Acute Abdomen Secondary to Ileal Perforation As the Initial Presentation of Advanced Müllerian Malignancy With Hepatic Metastasis: A Case Report

**DOI:** 10.7759/cureus.108962

**Published:** 2026-05-16

**Authors:** Shree Krishnamoorthy, Abirami Kailasam, Narayanan D Cunnigaiper

**Affiliations:** 1 General Surgery, Sri Ramachandra Institute of Higher Education and Research, Chennai, IND

**Keywords:** damage-control surgery, mullerian tumor, multi-disciplinary treatment, pelvic malignancy, rare clinical presentation, surgical acute abdomen

## Abstract

Acute abdomen in elderly postmenopausal women requires prompt evaluation to exclude life-threatening conditions such as bowel perforation, intra-abdominal sepsis, and malignancy. Advanced gynecological malignancies may involve the gastrointestinal tract, commonly through peritoneal dissemination or direct invasion. However, presentation as bowel perforation is rare and associated with significant morbidity.

A 61-year-old postmenopausal woman presented with diffuse abdominal pain and altered bowel habits. She was hemodynamically unstable with clinical features of sepsis. Imaging revealed a pelvic mass with suspected bowel involvement, pneumoperitoneum, and a hepatic lesion suggestive of metastasis. Emergency exploratory laparotomy demonstrated purulent peritoneal contamination and a sloughed ileal segment near the ileocecal junction, forming a mass with adjacent pelvic structures and a hepatic lesion suggestive of metastasis. Ileal resection with end ileostomy was performed. Histopathological examination of the bowel segment and liver lesion biopsy revealed metastatic carcinomatous deposits. Immunohistochemistry showed CK7+, PAX8+, WT1+, CK20-, and SATB2-, favoring a Müllerian origin. Further imaging demonstrated advanced disease with hepatic and vertebral metastases. The patient received combination chemotherapy with gemcitabine, carboplatin, and bevacizumab, achieving a near-complete metabolic response.

Bowel perforation as an initial presentation of pelvic malignancy is rare. This case highlights the importance of clinical judgment in managing an acute abdomen, especially when imaging findings are inconclusive. Immunohistochemistry plays a crucial role in identifying the primary tumor origin. Management requires a staged approach with emergency surgical intervention followed by multidisciplinary oncological treatment for optimal outcomes.

## Introduction

Acute abdomen in elderly and postmenopausal women represents a high-risk clinical scenario that often mandates urgent surgical intervention. While primary gastrointestinal etiologies are most frequently implicated, extraintestinal malignancies may rarely present with acute complications, thereby posing significant diagnostic and therapeutic challenges.

Müllerian malignancies encompass epithelial tumors arising from the ovary, fallopian tube, and primary peritoneum (serous, clear cell, mixed mullerian type). These tumors characteristically spread via transcoelomic dissemination, leading to peritoneal and omental involvement [1-3]. Gastrointestinal tract involvement is relatively common in advanced disease and typically occurs through serosal implantation or direct invasion, in which case immunohistochemistry [4] acts as an adjunct to diagnosis.

Bowel perforation in Müllerian malignancies is exceedingly rare, with only a few cases reported in literature [5-8]. When perforation does occur, it presents as an acute surgical emergency, often obscuring the underlying diagnosis and limiting opportunities for comprehensive oncological evaluation at initial presentation.

We report a rare case of Müllerian carcinoma (serous carcinoma) presenting as ileal perforation, highlighting the diagnostic complexity in emergency settings and underscoring the importance of considering gynecologic malignancies in the differential diagnosis of acute abdomen in postmenopausal women.

## Case presentation

A 61-year-old postmenopausal woman (P2L2, previous two lower+segment cesarean sections) with no significant comorbidities presented with a three-day history of diffuse abdominal pain and a 10-day history of altered bowel habits, without obstipation. On admission, she was tachycardic (115/min), tachypneic (22/min), and hypotensive (lowest recorded blood pressure 80/60 mmHg), with oxygen saturation of 98% on supplemental oxygen. Abdominal examination revealed mild distension with lower abdominal guarding. Per rectal examination, a fullness in the anterior wall with rectal mucosa intact, and per vaginal examination revealed a hard mass extending and involving the anterior and right lateral vaginal vault. Bilateral inguinal lymphadenopathy was noted (right greater than left).

**Figure 1:**
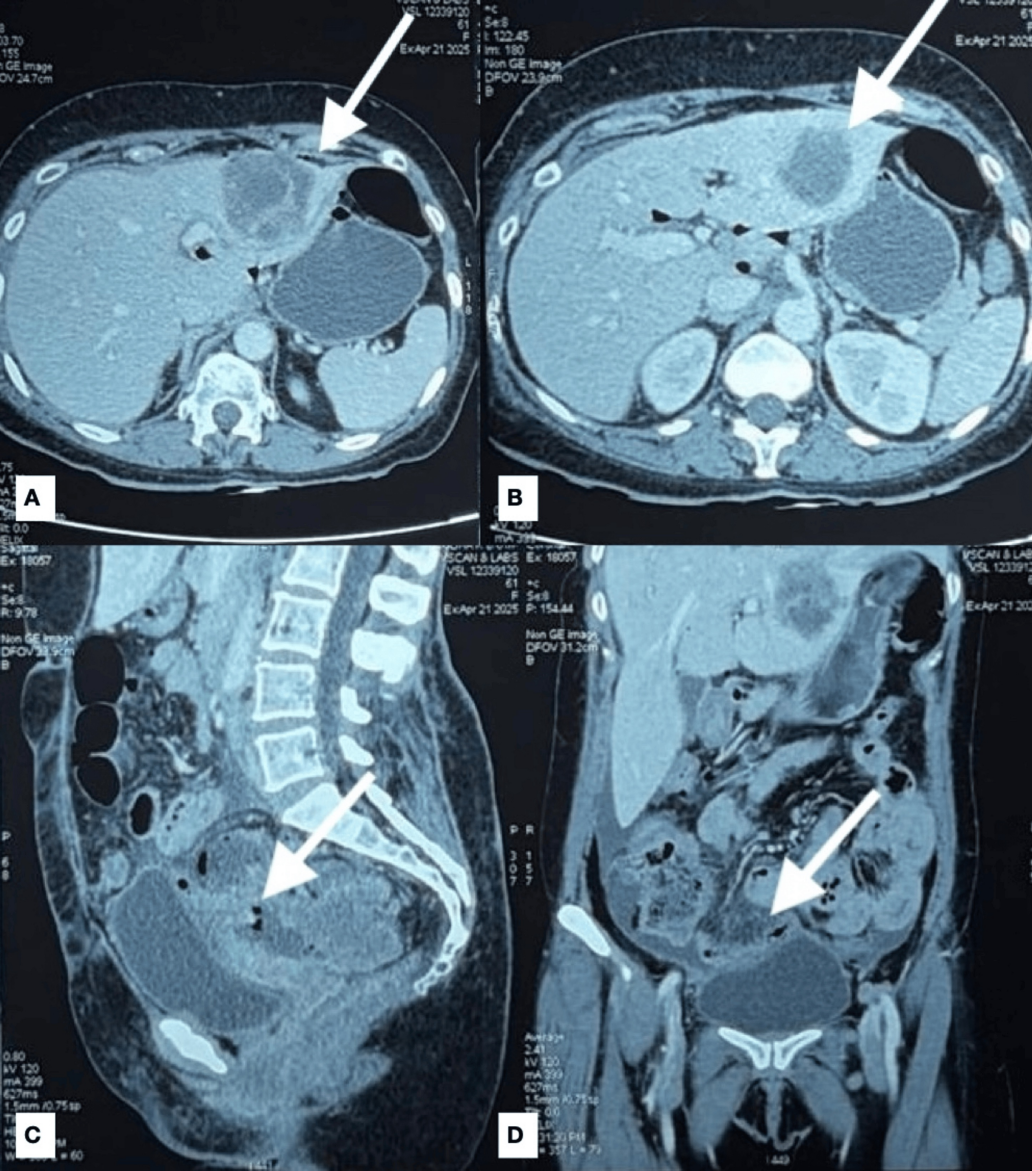
Preoperative contrast enhanced computed tomography of the abdomen A, B) Contrast-enhanced computed tomography of the abdomen in axial view showing a solitary large hepatic lesion occupying the left lobe of the liver, suggestive of metastatic disease; C) Contrast-enhanced computed tomography of the abdomen in Sagittal view showing a right adnexal mass lesion with localized air foci surrounding it - suggestive of pneumoperitoneum; D) Contrast-enhanced computed tomography of the abdomen in coronal view showing air foci in the pelvis surrounding the adnexal mass lesion, suggestive of pneumoperitoneum.

Laboratory investigations done upon presentation showed hemoglobin of 11.4 g/dL, leukocyte count of 4370/mm³, and elevated lactate levels on arterial blood gas analysis (Table 1), suggestive of sepsis. Contrast-enhanced computed tomography (Figure 1), done at a standalone scan center on the day of presentation, demonstrated a pelvic mass abutting bowel loops with suspected bowel infiltration, associated pneumoperitoneum, and localized infected pelvic collection, along with a hepatic lesion suspicious for metastasis.

**Table 1:** Preoperative laboratory parameters

Laboratory investigations	Patient parameters	Reference range
Hemoglobın	11.4 gms/dl	12-15 gms/dl
Total count	4370 cells/cumm	4000-11000 cells/cumm
Differential count – neutrophils	87.5%	45-70%
Platelet count	3.31 lakh/cumm	1.4-4.5 lak/cumm
Blood urea nitrogen	11 mg/dl	8-23 mg/dl
Creatinine	0.8 mg/dl	0.5-0.9 mg/dl
Sodium	135 mmol/L	136-145 mmol/L
Potassium	3.4 mmol/L	3.5-5.1 mmol/L
Chloride	102 mmol/L	98-107 mmol/L
Bicarbonate	16 mmol/L	22-29 mmol/L
Lactate (arterial blood gas)	2.2 mmol/L	0.20-1.80 mmol/L

**Figure 2:**
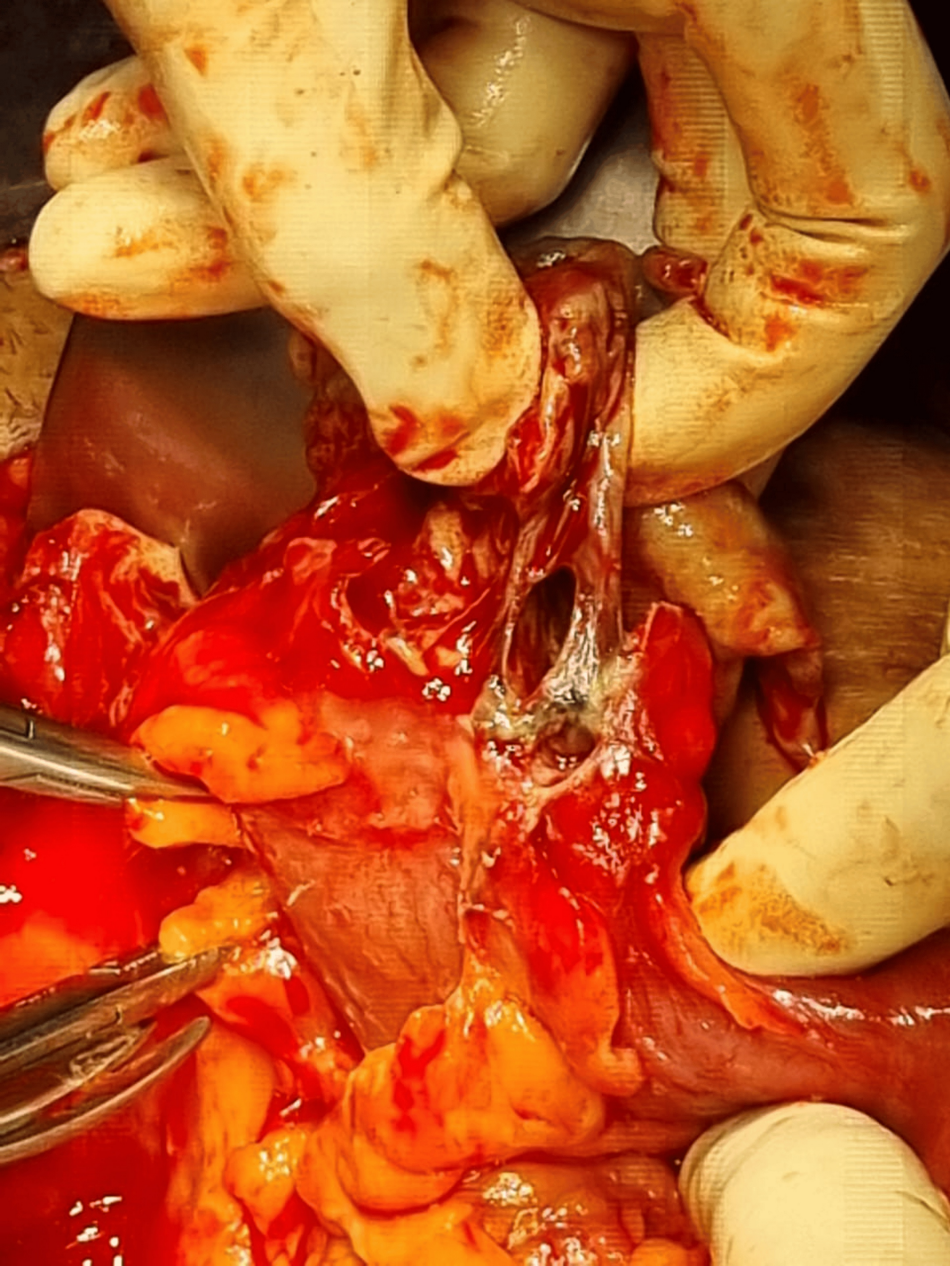
Intraoperative image - exploratory lapatoromy Intraoperative photograph demonstrating a perforation in the terminal ileum approximately 12 cm proximal to the ileocaecal junction, with surrounding inflammatory changes and areas of sloughed, nonviable tissue.

In view of hemodynamic instability, the patient was resuscitated with intravenous fluids and initiated on noradrenaline infusion. Emergency exploratory laparotomy revealed approximately 250 mL of purulent intraperitoneal collection. Dense adhesions were present in the pelvis, and a sloughed ileal segment was noted near the ileocecal junction (Figure 2), forming a mass with adjacent pelvic structures, including the uterus and bladder. Pelvic structures, including the rectum, uterus, ovaries, and fallopian tubes, could not be separately visualized due to dense adhesions. An oedematous and inflamed appendix was noted. A liver lesion measuring approximately 5 × 5 cm was also present. Overall, the peritoneal carcinomatosis index was noted to be 14/39. Ileal resection with end ileostomy and distal mucus fistula was performed in view of frozen pelvis, and peritoneal washings were obtained and sent for histopathological examination. Appendectomy was performed. The postoperative course was uneventful.

**Figure 3:**
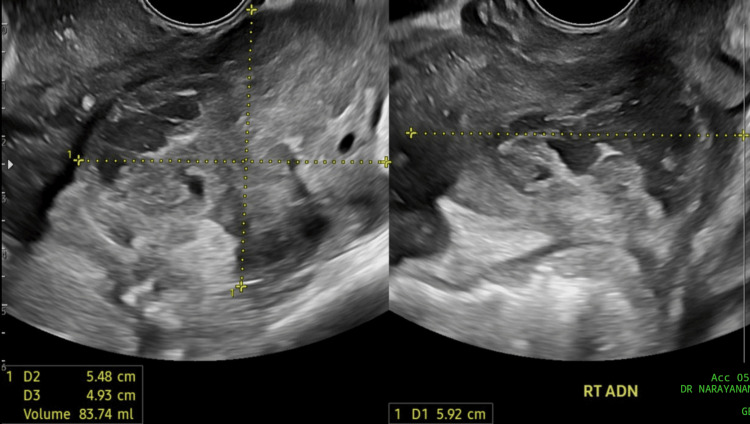
Postoperative transvaginal ultrasound Transvaginal ultrasound shows a relatively well defined heterogeneous mass lesion in the right adnexa.

**Figure 4:**
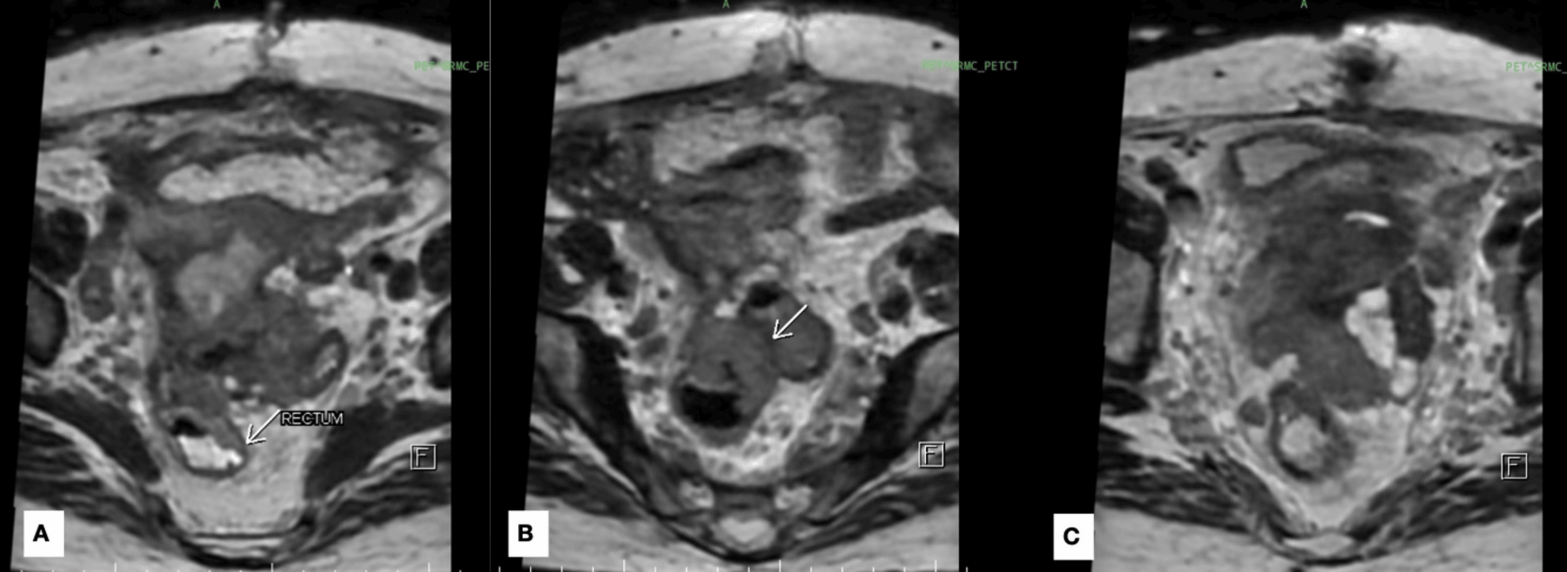
Postoperative magnetic resonance imaging of the pelvis A) Axial T2-weighted (T2W) image showing an ill-defined heterogeneous mass lesion arising from the upper rectum and sigmoid colon from 11 o'clock to 3 o'clock position. The mass lesion is noted extending anteriorly, infiltrating the posterior wall of the uterus; B, C) Axial T2W image showing a few of the adjacent small bowel loops adherent to the above-mentioned mass lesion.

**Figure 5:**
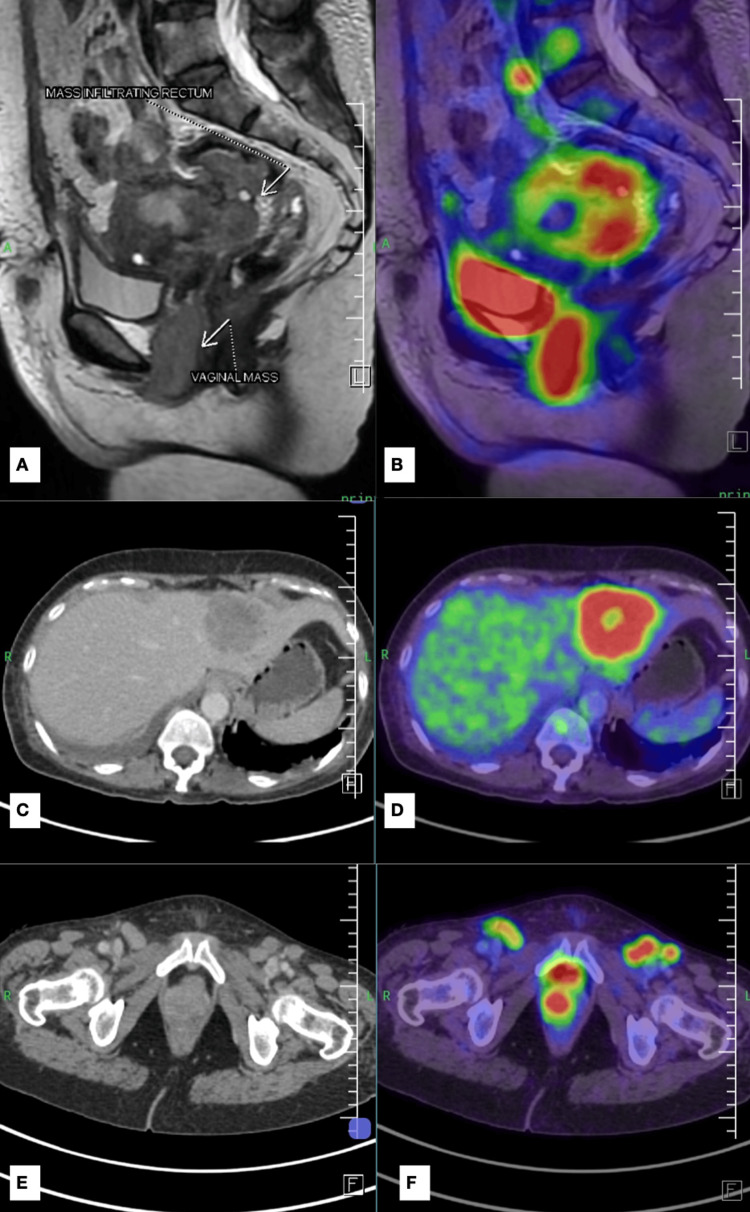
Postoperative positron emission tomography - computed tomography A) Sagittal T2-weighted (T2W) images show a relatively well-defined hyperintense mass lesion in the vagina. There is an ill-defined heterogeneous infiltrative mass lesion involving the upper 1/3rd of the rectum and distal sigmoid colon; B) These above-mentioned lesions show FDG avidity on the PET CT; C) Axial CT image showing a relatively well-defined heterogeneously enhancing lesion in the left lobe of the liver suggestive of metastasis; D) PET-CT image showing the same relatively well-defined heterogeneously enhancing lesion in the left lobe of the liver showing fluorodeoxyglucose (FDG) avidity suggestive of metastasis; E) Axial CT images showing mass lesion in the vagina with multiple enlarged bilateral inguinal lymph nodes; F) Axial PET CT images show FDG-avid mass lesion in the vagina and multiple FDG avid bilateral inguinal lymph nodes

Further postoperative imaging with transvaginal ultrasonography (Figure 3) showed a predominantly solid heterogeneous right adnexal mass of size 5.5x4.9x5.9 cm with loss of fat planes with the uterus - Ovarian Adnexal Reporting and Data System 4 (O-RADS 4). Positron emission tomography - computed tomography with magnetic resonance imaging screening (Figure 4 A-C) further demonstrated a fluorodeoxyglucose (FDG)-avid pelvic lesion with infiltration (Figure 5A-B) into the sigmoid and upper rectum with vaginal involvement, liver metastasis, extensive lymphadenopathy (Figure 5C-F) and suspicious foci of metastasis in D11 and D9 vertebrae, suggestive of malignancy arising from the rectum with large extramural component. Blood investigations revealed a cancer antigen 125 of 162.10, carcinoembryonic antigen of 0.37, and carbohydrate antigen 19-9 of 57.3 (Table 2).

**Table 2:** Tumor markers

Laboratory investigations	Patient value	Reference range
Cancer antigen 125 (CA -125)	162.10 U/mL	<35 U/mL
Carbohydrate antigen 19-9 (CA 19-9)	57.30 U/mL	<34 U/mL
Carcinoembryonic antigen (CEA)	0.37 ng/ml	Non smoker <3.8 ng/ml/ Smoker <5.5 ng/ml

Histopathological examination of the resected ileal bowel specimen demonstrated metastatic carcinomatous deposits on the serosal surface with extensive necrosis and perforation. Immunohistochemistry showed positivity for CK7, PAX8, and WT1, and negativity for CK20 and SATB2 (Table 3), favoring a primary malignancy of the female genital tract, probably ovarian in origin (serous ovarian carcinoma). Peritoneal washings showed an inflammatory process and was negative for malignancy. The appendix showed features of acute suppurative appendicitis. Postoperative USG-guided liver biopsy revealed malignant neoplastic cells arranged in glandular patterns with necrosis, consistent with metastatic disease. 

**Table 3:** Immunohistorchemistry status on intraoperative ileal specimen sent for histopathology

Immunohistochemistry marker	Full form	Status in patient
CK7	Cytokeratin 7	Positive
CK20	Cytokeratin 20	Negative
PAX8	Paired box gene 8	Positive
WT1	Wilms tumor 1	Positive
SATB2	Stabilin-2	Negative

The case was discussed in a multidisciplinary tumor board and managed as advanced Müllerian carcinoma with metastasis in view of histopathological and positron emission tomography - computed tomography findings [9]. As the peritoneal carcinoma index was noted to be 14/39, the patient was advised to undergo chemotherapy. The patient received systemic chemotherapy (Gemcitabine, Carboplatin, Bevacizumab) as per current consensus recommendations [10,11], last dose completed on 31/1/2026, and with partial metabolic response noted as per the PET Response Criteria in Solid Tumors (PERCIST) on follow-up positron emission tomography - computed tomography dated 16/2/2026. At present, she is on maintenance therapy with bevacizumab. 

## Discussion

This case highlights an uncommon presentation of advanced pelvic malignancy [12,13] manifesting as an acute abdomen due to bowel perforation [5-7], with initial imaging suggestive of an abscess secondary to malignant perforation. Gastrointestinal involvement in gynecological malignancies typically occurs via peritoneal seeding or direct invasion [2]; however, full-thickness bowel necrosis and perforation are rarely reported [5-8].

In the above-discussed case, determining the primary site of malignancy was challenging due to the emergent nature of presentation, where immediate surgical intervention took precedence over comprehensive oncological workup. Differentiation between primary colorectal carcinoma and metastatic Müllerian carcinoma is crucial, as management differs significantly. In this particular case, imaging features are suggestive of infiltration into the rectum. While Müllerian tumors typically involve the female genital tract, their presentation can be extra-genital, including the rectum. In this case, immunohistochemistry [4] with the CK7+ and CK20- phenotype served as a definitive diagnostic marker, confirming Müllerian origin (PAX8+, WT1+, CK7+, CK20−), directing treatment plans specific to gynecological epithelial carcinoma, despite elevated serum markers (CA19-9). The peritoneal washings showed no evidence of malignant cells, and the appendix was likely inflamed secondary to the peritonitis following perforation. The presence of synchronous liver metastasis corresponds to advanced-stage disease as per the International Federation of Gynecology and Obstetrics (FIGO) classification [12,13].

Bowel perforation in malignancy carries high morbidity and mandates urgent surgical intervention. Damage-control surgery with resection and diversion, as performed here, aligns with international recommendations for intra-abdominal sepsis management [3]. Equal importance was assigned to postoperative evaluation of the patient to ensure multidisciplinary treatment and guided systemic chemotherapy.

This case underscores the critical role of clinical assessment in managing acute abdomen, cautioning against over-reliance on imaging. Here, a missed clinical diagnosis of perforation could have led to significant morbidity and mortality. Rare case reports have documented ovarian tumors presenting as an acute abdomen due to rupture or perforation [5-8]. Furthermore, it illustrates that advanced gynecological malignancies in the elderly can rarely present as bowel perforations and require a staged, individualized approach in emergency settings.

This report has limitations. The absence of an extended immunohistochemical panel, particularly tumor protein p53, estrogen receptor (ER), and progesterone receptor (PR), limits specific characterization of tumor biology. The serum Ca 19-9 elevation (57.3U/ml), although explained by its documented association as a biomarker with ovarian malignancies [14], may reduce specificity in distinguishing the primary origin. Management decisions were influenced by the acute presentation and patient factors, including advanced age and hemodynamic instability, which necessitated an initial damage control surgery rather than definitive multivisceral resection. Definitive treatment planning was subsequently guided after multidisciplinary evaluation, with chemotherapy being considered in contrast to complete oncological resection in view of disease burden, high Peritoneal Carcinomatous Index of 14/39, and patient status. Despite these limitations, this case highlights an atypical presentation of Müllerian carcinoma as bowel perforation requiring situational awareness and a high index of clinical suspicion from the operating surgeon to decide on damage control surgery in cases of hemodynamic instability, or mutivisceral resection [5-8], if the patient's hemodynamic status permits. 

## Conclusions

Pelvic malignancies may rarely present as an acute abdomen due to bowel perforation, posing significant diagnostic and therapeutic challenges. Prompt resuscitation, timely surgical intervention, and a high index of suspicion are essential.

Immunohistochemistry plays a pivotal role in identifying the primary tumor origin in complex presentations, as initial serum markers and radiographic imaging yielded confounding results (ORADS 4 on USG and PET CT suggestive of rectal malignancy). With discordant imaging findings, the key to diagnosis lay in the histopathological markers, thus preventing the patient from undergoing further invasive procedures.

A staged approach involving damage-control surgery followed by multidisciplinary oncological management tailored to the patient's specific condition is crucial for optimizing outcomes. In the presented case, post-damage-control surgery, conventional resection was deferred after taking multiple patient and disease factors into account. 

Recognition of such atypical presentations is important, as early diagnosis and coordinated care, along with appropriate intraoperative tissue sampling, are essential to guide further oncologic management and can significantly influence prognosis even in advanced-stage disease. 
